# Effectiveness of a Personalized, Chess-Based Training Serious Video Game in the Treatment of Adolescents and Young Adults With Attention-Deficit/Hyperactivity Disorder: Randomized Controlled Trial

**DOI:** 10.2196/39874

**Published:** 2023-04-24

**Authors:** María Rodrigo-Yanguas, Marina Martín-Moratinos, Carlos González-Tardón, Fernando Sanchez-Sanchez, Ana Royuela, Marcos Bella-Fernández, Hilario Blasco-Fontecilla

**Affiliations:** 1 Servicio de Psiquiatría, Hospital Universitario Puerta de Hierro Majadahonda Majadahonda Spain; 2 Facultad de Medicina, Universidad Autónoma de Madrid Madrid Spain; 3 Universidad de Diseño y Tecnología Madrid Spain; 4 R&D Department, Hogrefe TEA Ediciones Madrid Spain; 5 Biostatistics Unit Hospital Universitario Puerta de Hierro Majadahonda Madrid Spain; 6 Consorcio de Investigación Biomédica en Red: Epidemiología y Salud Pública Madrid Spain; 7 Facultad de Psicología, Universidad Autónoma de Madrid Madrid Spain; 8 Departamento de Psicología, Universidad Pontificia de Comillas Madrid Spain; 9 Consorcio de Investigación Biomédica en Red: Salud Mental Madrid Spain; 10 Ita Mental Health Madrid Spain

**Keywords:** attention-deficit hyperactivity disorder, ADHD, serious video games, cognitive training, chess, video game, teen, young adult, game, intervention, treatment, emotional, control, regulation, attention, school, function, symptom

## Abstract

**Background:**

Compared with traditional approaches, gaming strategies are promising interventions for the treatment of attention-deficit/hyperactivity disorder (ADHD). We developed a serious game, The Secret Trail of Moon (TSTM), for ADHD treatment.

**Objective:**

The main objective of this clinical trial was to demonstrate the effectiveness of an add-on, either TSTM or Therapeutic Chess (TC), in previously optimally drug-titrated, clinically stable patients with ADHD.

**Methods:**

This study is a prospective, unicentric, randomized clinical trial in clinically stable patients with ADHD, aged 12 to 22 years. The TSTM (n=35) and TC groups (n=34) performed 12 weekly sessions of their respective treatments. The control group (CG) patients (n=35) were called by phone every week, but they received no cognitive intervention. The primary end point was the change from baseline to end point in the parent “Behavior Rating Inventory of Executive Function-2” (BRIEF-2; patients’ parents) in the per-protocol population (31 serious videogame: 24 TC and 34 CG).

**Results:**

Our study failed to probe clear-cut improvements in the global score of the BRIEF-2. However, the TC group showed improvements in measures of emotional control, emotional regulation, and inattention. The TSTM group showed improvements in measures of emotional regulation, inattention, and school context.

**Conclusions:**

TSTM and TC did not improve executive function symptoms, but they improved ADHD symptomatology related to emotional regulation. Further studies with bigger samples are required to confirm these preliminary findings.

**Trial Registration:**

ClinicalTrials.gov NCT04355065; https://clinicaltrials.gov/ct2/show/NCT04355065

## Introduction

Attention-deficit/hyperactivity disorder (ADHD) is the most frequent neurobehavioral disorder, with a worldwide prevalence of 4% to 8% [1,2] and a relative risk of 3:1 against males [3]. ADHD is multicausal; about 70% is due to genetic factors, and the other 30% is due to environmental factors [4]. It is characterized by the presence of 3 core symptoms: inattention, hyperactivity, and impulsivity. These symptoms must be present in 2 or more contexts and interfere with daily functioning [5,6]. Furthermore, patients with ADHD experience a main deficit in prefrontal and anterior areas [7,8], the inferior parietal lobe, supramarginal gyrus, basal ganglia, and premotor area. Deficiencies in both executive functions [9-13] and emotional intelligence [4,14] are critically involved with ADHD.

Reference guides recommend multimodal treatment for ADHD: pharmacological intervention and behavioral therapy—behavioral training (BT) for parents and organizational training for adolescents and adults or a combination [4,15,16]. Unfortunately, multimodal treatment is not always sufficient for the treatment of ADHD symptoms [17,18]. The literature on the efficacy of cognitive behavioral therapy in ADHD is still equivocal, although some trials focused on organizational training in adolescents and adults showed promising results [19-21]. Cognitive behavioral therapy is not recommended in children younger than 10 years. Accordingly, novel strategies are warranted [22-24]. For instance, BTs are the most recommended psychological treatments for ADHD [25]. BTs are aimed at stimulating executive functions, among others [26]. Unfortunately, BTs are financially expensive and may be monotonous for adolescent patients [27]. To address these handicaps, some therapists have begun to use gaming strategies, such as (1) traditional board games like chess [28-30] and Go [31], (2) 2D video games [32-36], and (3) virtual reality serious video games [37-39]. Indeed, in a recent clinical trial including 857 children with ADHD, the patients randomized to Akili Interactive—the first videogame that can be prescribed to patients with ADHD—improved more than those who were randomized to the digital control intervention group [40]. Compared with traditional therapies, gaming strategies have several advantages, such as increased engagement, lower costs [28,41-44], and improved performance [45].

In this context, we developed The Secret Trail of Moon (TSTM), a virtual reality chess-based serious video game aimed at improving attention, impulse control, visuospatial capacity, planning, self-regulation, working memory, reasoning, and cognitive flexibility. TSTM is innovative, motivating, and customizable; it is fun, easy to understand and play, displays enjoyable graphics, and has adequate duration for most participants [39].

This study is aimed at comparing the effectiveness of 2 cognitive gaming strategies in previously optimally drug-titrated, clinically stable patients with ADHD: (1) electronic Therapeutic Chess (TC); and (2) TSTM. Recently, we published the protocol study of our add-on clinical trial [46]. Basically, the TC group received chess lessons and had to solve both traditional chess exercises (related to the video tutorial for each week) and TC exercises. These exercises use the pieces and board but can be done without knowing how to play chess [46]. The control group (CG) received no specific cognitive intervention. We hypothesized that patients randomized to either TSTM or TC, compared to the CG group, would improve in executive functions and ADHD core symptoms.

## Methods

### Study Design

The study protocol (registered on the ClinicalTrials website: NCT04355065) is published elsewhere [46], but it is basically a prospective, unicentric, randomized, non-equality trial in 105 patients with ADHD. All participants were clinically stable before baseline. Subjects were randomized into 3 groups: TC group (electronic cognitive training with TC), TSTM group (cognitive training using TSTM), and CG (patients were called by phone every week, but they received no cognitive intervention). The allocation ratio was equal in all 3 groups (n=35 per branch).

### Ethics Approval

The study was approved by the Ethics Committee of the Puerta de Hierro University Hospital in Majadahonda on December 2, 2019 (PI 187/19).

### Study Population

The clinical trial included children, adolescents, and young adults between the ages of 12 and 22 years (mean age 14.38, SD 2.26 years). The minimum participation age was 12 because virtual reality is discouraged for children younger than 12 years. The maximum age was 22 because brain maturation continues until that age, approximately [47,48]. Furthermore, adolescent patients admitted in Child and Adolescent Psychiatry units are progressively referred to be followed up in Adult Psychiatry services, which means that patients up to the age of 20 (and occasionally 22) years are already followed up in Child and Adolescent Psychiatry units [46]. Inclusion criteria were as follows: adolescents or young adults between the ages of 12 and 22 years with a diagnosis of ADHD and clinically stable on any medication related to ADHD, meaning that participants had a consistent drug prescription and were not allowed to change it during their participation; however, during the trial, 5 participants changed their medication, another 5 participants increased their medication dosages, another 5 participants decreased their medication dosages, and medication was removed for another participant. Exclusion criteria were the following: comorbidities with autism or intellectual disability, epilepsy, or another severe clinical condition; plans to begin another clinical treatment or moving out during the following 3 months; and risk of suicide [46]. A total of 105 patients (35 per group) were recruited for the clinical trial, of which 47 (45.2%) had an inattentive ADHD subtype and 57 (54.8%) had a mixed ADHD subtype.

The participants were recruited between December 18, 2019, and November 16, 2020. The last evaluation was performed on February 6, 2021.

### Randomization and Masking

To warrant clinical trial randomization, AR generated a sequence of random numbers through Epidat 4.2 software (Dirección Xeral de Saúde Pública, Xunta de Galicia). The 105 patients were randomly assigned to the 3 groups by HBF following the randomized sequence. Each randomization number was introduced inside a closed, opaque envelope. When a patient came to the hospital to participate in the trial, the inclusion and exclusion criteria were applied to them (one participant was excluded then); the patients and their parents received and signed informed consents, and after the first assessment (D0), each participant received and opened the envelope corresponding to their inclusion number, and the group assigned to that number.

### Study Interventions

#### Visits

The number of visits depended on the group. The TSTM group made 15 in-person visits over 3 months: pre-inclusion visit, inclusion visit (D0), 12 cognitive treatment visits (1 per week) with TSTM, and one final visit (D90).

The TC and CG groups made 3 in-person visits over 3 months: pre-inclusion visit, inclusion visit (D0), and postassessment visit (D90). A total of 12 remote (web- or phone-based) visits were also made during the 3 months.

#### Interventions

A more detailed description of the intervention groups can be found elsewhere [46]. Basically, the interventions were different for each group, as follows:

- Cognitive intervention with TSTM (TSTM group): TSTM was constructed for 5 different cognitive training mechanisms: smasher, enigma, teka-teki, kuburi, and chess. Each session was divided into 3 blocks (2 blocks for training into 2 different game mechanics and a chess training block). Sessions were personalized in the sense that difficulty levels were adjusted, taking into account individual performance.
- Cognitive intervention with TC (TC group): in the initial visit, each patient received a flash drive containing the 12 training sessions in TC. Every session was divided into 3 parts: video tutorials, traditional chess exercises, and TC exercises.
- Control group intervention (CG group): each patient received 12 phone communications (1 per week). In these communications, they were asked about medication usage, mood, and school performance, among other issues.

### Study Outcomes

#### Primary Outcome

The primary end point was the change from baseline to the end of treatment in the “Behavior Rating Inventory of Executive Function-2” (BRIEF-2) questionnaire filled out by the patients’ parents [49,50]. This scale consists of 63 items and 4 main indicators: emotional regulation, cognitive regulation, behavioral regulation, and executive function global index. The design of TSTM is based on cognitive training and task and aimed at improving executive functions [39]. Additionally, TC has shown a certain effect on executive functions [28].

#### Secondary and Safety Outcomes

Secondary measures were the ATENTO questionnaire [51], with versions for patients and for parents; the emotional intelligence inventory by the BarOn model of Emotional Quotient Inventory (BarOn EQ-i: Youth Version), with a version for younger people [52]; the ADHD rating scale V (ADHD-V); the quantitative scale by Swanson, Nolan, and Pelham (SNAP-IV) [53]; the Conners’ Parent Rating Scale (CPRS-HI) [54]; and the Conners’ Continuous Performance Test 3 (CPT-3) for computers [55]. In addition, the TSTM group filled out the Udvalg für Kliniske Undersolgester (UKU) questionnaire [56,57].

### Statistics

#### Sample-Size Calculation

Sample sizes were calculated for 80% of statistical power, assuming 2-tailed Student *t* tests for comparison between groups, with a 1:1:1 proportion, assuming a 20% loss. In each group, 35 patients were included (a total of 105 patients).

#### Analyses

Analyses were performed for two types of populations: analyses for treatment intention (ATI) and analysis per protocol (PP). In ATI, data were analyzed according to the assigned treatment for each patient. In PP, data were analyzed according to the treatment that each patient completed.

A descriptive analysis was performed for qualitative variables using absolute and relative frequencies. For quantitative variables, the median and the quartiles 1 and 3 were used as descriptive statistics.

To compare the groups TSTM versus CG and TC versus CG, 2 series of analyses were performed in parallel. To compare the basal and final differences, the Kruskal-Wallis test was performed, as the normality assumption for ANOVAs and *t* tests was generally violated. The signification level was fixed at *P=.*10 mainly due to the pandemic situation and the early stage of this trial [58]. Subsequently, for those variables reaching statistical significance in Kruskal-Wallis tests, pairwise Mann-Whitney tests were performed to compare pairs of experimental groups. The effect sizes were estimated through ε^2^ for Kruskal-Wallis tests and η^2^ for Mann-Whitney tests [59]. The statistical package SPSS 26 (IBM Corp) was used to perform the tests. Ultimately, the study was powered to detect an effect of Cohen *d*≥0.52 in two-way comparisons.

## Results

### Patient Inclusion and Randomization

**Figure 1:**
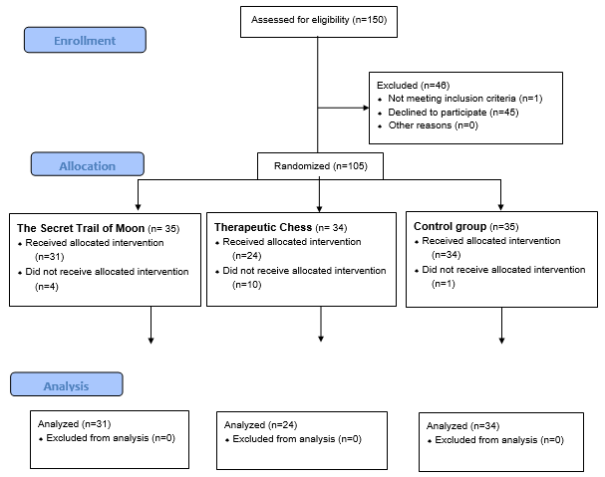
CONSORT Flow Diagram for the number of participants in each stage of the trial.

A total of 105 patients agreed to participate in the trial and signed a reported consent form. Ultimately, 1 patient was excluded for having a previously unreported history of epilepsy. The other 104 patients were randomized to one of 3 groups: TSTM (n=35), TC (n=34), and CG (n=35). Figure 1 shows a flow diagram describing in detail the number of participants in each trial stage.

### Baseline Characteristics

In the PP population at D90, participants were male in 71.9% (64/89) of the cases (TSTM group: 27/31, 87.1%; TC group: 19/24, 79.2%; and CG: 18/34, 53.1%). The mean ages for the groups were 14.7 (SD 2.4) for TSTM, 13.8 (SD 1.9) for TC, and 14.8 (SD 2.4) for CG. Table 1 and Table 2 show all the sociodemographic and baseline variables.

**Table 1:** Sociodemographic and baseline variables for analysis for treatment intention (ATI). Italicized *P* values are significant.

Characteristics		ATI								
		TSTM^a^ (n=35)	TC^b^ (n=34)	CG^c^ (n=35)	Chi-square (df)		Kruskal-Wallis (df)		*P* value	
Gender (female), n (%)		8 (22.86)	9 (26.47)	16 (45.71)	4.866 (2)		N/A^d^		*.09*	
Age (years), mean (SD)		14.7 (2.4)	13.8 (1.9)	14.6 (2.4)	N/A		3.171 (2)		.20	
Birthplace (Spain), n (%)		34 (97.14)	29 (85.29)	34 (97.14)	5.188 (2)		N/A		*.08*	
**Adoption, n (%)**						1.992 (4)		N/A		.74
	Adopted	2 (5.71)	2 (5.88)	2 (5.71)						
	Shelter home	0 (0)	0 (0)	1 (2.86)						
**ADHD^e^ diagnosed, n (%)**						1.771 (2)		N/A		.41
	Inattentive	19 (54.29)	14 (41.18)	14 (40)						
	Combined	16 (45.71)	20 (58.82)	21 (60)						
**Medication, n (%)**						17.016 (12)		N/A		.15
	Elvanse	24 (68.57)	24 (70.59)	26 (74.29)						
	Medikinet	7 (20)	2 (5.88)	3 (8.57)						
	Equasym	3 (8.57)	1 (2.94)	2 (5.71)						
	Rubricon	1 (2.86)	2 (5.88)	2 (5.71)						
	Stratera	0 (0)	3 (8.82)	0 (0)						
	Intuniv	0 (0)	1 (2.94)	0 (0)						
	Concerta	0 (0)	0 (0)	2 (5.71)						

^a^TSTM: The Secret Trail of Moon.

^b^TC: Therapeutic Chess.

^c^CG: control group.

^d^N/A: not applicable.

^e^ADHD: attention-deficit/hyperactivity disorder.

**Table 2:** Sociodemographic and baseline variables for analysis per protocol (PP). Italicized *P* values are significant.

Characteristics		PP								
		TSTM^a^ (n=31)	TC^b^ (n=24)	CG^c^ (n=34)	Chi-square (df)		Kruskal-Wallis (df)		*P* value	
Gender(female), n (%)		4 (12.9)	5 (20.8)	16 (47.1)	10.222 (2)		N/A^d^		*.006*	
Age (years), mean (SD)		14.3 (2.246)	13.81 (1.939)	14.83 (2.479)	N/A		2.310 (2)		.31	
Birthplace (Spain), n (%)		30 (96.8)	21 (87.5)	33 (97.1)	2.938 (2)		N/A		.23	
**Adoption, n (%)**						2.316 (4)		N/A		.68
	Adopted	1 (3.2)	2 (8.3)	2 (5.9)						
	Shelter home	0 (0)	0 (0)	1 (2.9)						
**ADHD^e^ diagnosed, n (%)**						1.800 (2)		N/A		.41
	Inattentive	17 (54.8)	11 (45.8)	13 (38.2)						
	Combined	14 (45.2)	13 (54.2)	21 (61.8)						
**Medication, n (%)**						16.8 (10)		N/A		.08
	Elvanse	21 (67.74)	15 (62.5)	26 (76.47)						
	Medikinet	6 (19.35)	2 (8.33)	3 (8.82)						
	Equasym	3 (9.68)	1 (4.17)	2 (5.88)						
	Rubricon	0 (0)	2 (8.33)	2 (5.88)						
	Stratera	0 (0)	3 (12.5)	0 (0)						
	Concerta	0 (0)	0 (0)	2 (5.88)						

^a^TSTM: The Secret Trail of Moon.

^b^TC: Therapeutic Chess.

^c^CG: control group.

^d^N/A: not applicable.

^e^ADHD: attention-deficit/hyperactivity disorder.

The BRIEF-2 questionnaire shows that the TSTM group performed better than the TC and CG groups in “working memory,” “planification and organization,” and “cognitive regulation" scales, the groups being approximately equal in the rest of the BRIEF-2 scales (Tables S1 and S2 in Multimedia Appendix 1 present descriptive baseline characteristics).

For both primary and secondary outcomes, we calculated individual pre-post differences and used these differences as the dependent variables in our tests.

### Primary Outcomes

The goal of this study was ultimately to compare the changes between D0 and D90 in every group as well as the differences among groups. The main measurement for testing the differences were the BRIEF-2 scores, according to the indications already presented elsewhere [46]. The groups were compared through Kruskal-Wallis tests.

ATI showed improvements for the TC group compared to the CG group in “emotional control” and “emotional regulation” (*P*<.10) scores. PP also showed a significant difference in “emotional control” between the TC and CG groups (*P*<.10). None of the rest of the comparisons reached a statistical significance. All tests and significance levels are shown in Tables 3 and Table 4.

**Table 3:** Kruskal-Wallis tests for primary outcomes (Behavior Rating Inventory of Executive Function-2 [BRIEF-2] scales).

Characteristics	ATI^a^ (n=100)				PP^b^ (n=89)		
	Kruskal-Wallis	ε^2^	*P* value	Kruskal-Wallis		ε^2^	*P* value
Inhibition	0.693	0.007	.71	1.039		0.012	.60
Self-supervision	0.255	0.003	.88	0.201		0.002	.90
Flexibility	1.425	0.014	.49	1.891		0.021	.39
Emotional control	4.388	0.044	.11	4.417		0.050	.11
Initiative	0.114	0.001	.95	0.053		0.001	.97
Working memory	0.610	0.006	.74	0.025		<0.001	.99
Planning and organization	0.617	0.006	.73	0.233		0.003	.89
Task supervision	3.055	0.031	.22	2.953		0.034	.23
Material organization	0.301	0.003	.86	0.026		<0.001	.99

^a^ATI: analyses for treatment intention.

^b^PP: per protocol.

**Table 4:** Kruskal-Wallis tests for BRIEF-2 Indexes.

Characteristics	ATI^a^ (n=100)				PP^b^ (n=89)		
	Kruskal-Wallis	ε^2^	*P* value	Kruskal-Wallis		ε^2^	*P* value
Behavioral regulation	0.496	0.005	.78	1.286		0.015	.53
Emotional regulation	3.090	0.031	.21	2.920		0.033	.23
Cognitive regulation	1.808	0.018	.41	0.718		0.008	.70
Global executive function	1.435	0.014	.49	0.926		0.011	.63

^a^ATI: analyses for treatment intention.

^b^PP: per protocol.

### Secondary Outcomes

Analyses for secondary outcomes were performed in the PP population. Table 5 shows all the tests and significance levels for the secondary outcomes. Table 5 highlights those variables that reached significance in Kruskal-Wallis tests and the pairwise comparisons between the three trial groups.

**Table 5:** Kruskal-Wallis tests for secondary outcomes (only for PP). The italicized *P* values are significant.

Characteristics	Kruskal-Wallis	ε^2^	*P* value
SNAP-IV^a^: inattention	2.859	0.032	.24
SNAP-IV: hyperactivity	0.231	0.003	.89
SNAP-IV: total	1.218	0.014	.54
ATENTO (self-report): inattention	2.262	0.026	.32
ATENTO (self-report): hyperactivity	1.300	0.015	.52
ATENTO (self-report): attentional control	1.785	0.020	.41
ATENTO (self-report): behavioral regulation	0.975	0.011	.61
ATENTO (self-report): emotional regulation	3.507	0.040	.17
ATENTO (self-report): working memory	0.439	0.005	.80
ATENTO (self-report): flexibility	2.137	0.024	.34
ATENTO (self-report): planning and organization	2.776	0.032	.25
ATENTO (self-report): time orientation	7.170	0.081	*.03*
ATENTO (self-report): behavioral problems	1.942	0.022	.38
ATENTO (self-report): sleeping problems	0.741	0.008	.69
ATENTO (self-report): family context	0.750	0.009	.69
ATENTO (self-report): school context	5.283	0.060	*.07*
ATENTO (self-report): social context	0.431	0.005	.81
ATENTO (family): inattention	0.219	0.002	.90
ATENTO (family): hyperactivity	0.272	0.003	.87
ATENTO (family): attentional control	1.236	0.014	.54
ATENTO (family): behavioral regulation	0.380	0.004	.83
ATENTO (family): emotional regulation	0.799	0.009	.67
ATENTO (family): working memory	1.337	0.015	.51
ATENTO (family): flexibility	2.902	0.033	.23
ATENTO (family): planning and organization	2.563	0.029	.28
ATENTO (family): time orientation	3.521	0.040	.17
ATENTO (family): behavioral problems	3.097	0.035	.21
ATENTO (family): sleeping problems	1.146	0.013	.56
ATENTO (family): family context	2.640	0.030	.27
ATENTO (family): social context	2.230	0.025	.33
EQ-i:YV^b^: positive impression	5.826	0.066	*.05*
EQ-i:YV: mood	2.307	0.026	.32
EQ-i:YV: total emotional intelligence	3.827	0.043	.15
EQ-i:YV: intrapersonal	0.696	0.008	.71
EQ-i:YV: interpersonal	5.002	0.057	*.08*
EQ-i:YV: adaptability	1.909	0.022	.39
EQ-i:YV: stress management	2.772	0.032	.25
CPT-3^c^ response style	2.905	0.033	.23
CPT-3 detectability	5.483	0.062	*.06*
CPT-3 omissions	2.552	0.029	.28
CPT-3 commissions	2.369	0.027	.31
CPT-3 perseverations	5.207	0.059	*.07*
CPT-3 reaction times (hits)	4.139	0.047	.13
CPT-3 reaction time variability (hits)	6.465	0.073	*.04*
CPT-3 variability	3.340	0.038	.19
CPT-3 block change	7.605	0.086	*.02*
CPT-3 inter stimulus change	9.007	0.102	*.01*
CPRS^d^	0.884	0.010	.64

^a^SNAP: Swanson, Nolan, and Pelham.

^b^EQ-i:YV Emotional Quotient Inventory: Youth Version.

^c^CPT-3: Continuous Performance Test 3.

^d^CPRS: Conners’ Parents Rating Scale.

**Table 6:** Pairwise Mann-Whitney tests for those variables that reached significance in Tables 3 to 5. The italicized *P* values are significant.

Characteristics	TSTM^a^ vs TC^b^					TSTM vs CG^c^					TC vs CG			
	Mann-Whitney test	η^2^	*P* value	Direction of difference	Mann-Whitney test		η^2^	*P* value	Direction of difference	Mann-Whitney test		η^2^	*P* value	Direction of difference
ATENTO (self-report): time orientation	219	0.068	*.01*	TC>TSTM	519		<0.001	.94	—^d^	273		0.058	*.02*	TC>CG
ATENTO (self-report): school context	315	0.007	.43	—	357.5		0.055	*.03*	TSTM>CG	328.5		0.023	.16	—
EQ-i:YV^e^: positive impression	261	0.034	*.08*	TC>TSTM	351		0.060	*.02*	CG>TSTM	402		0.001	.78	—
EQ-i:YV: interpersonal	340	0.001	.73	—	397.5		0.032	*.09*	CG>TSTM	286.5		0.048	.04	—
Conners’ CPT-3^f^ detectability	219	0.068	*.01*	TC>TSTM	428.5		0.018	.20	—	356.5		0.011	.33	—
Conners’ CPT-3 perseverations	234	0.056	*.03*	TC>TSTM	427.5		0.019	.19	—	348.5		0.015	.25	—
Conners’ CPT-3 reaction time variability (hits)	224	0.063	*.02*	TC>TSTM	402.5		0.029	.11	—	335		0.019	.19	—
Conners’ CPT-3 block change	290	0.017	.22	—	370.5		0.047	*.04*	CG>TSTM	265.5		0.064	.02	CG>TC
Conners’ CPT-3 inter stimulus change	215.5	0.071	*.01*	TC>TSTM	334		0.071	*.01*	CG>TSTM	359		0.010	.35	—

^a^TSTM: The Secret Trail of Moon.

^b^TC: Therapeutic Chess.

^c^CG: control group.

^d^Not applicable.

^e^EQ-i:YV Emotional Quotient Inventory: Youth Version.

^f^CPT-3: Continuous Performance Test 3.

According to the protocol study of our add-on clinical trial [46], scores on ADHD-5, CPRS, SNAP-IV, ATENTO, BarOn EQ-i:YV, and CPT-3 tests are considered secondary end point analyses. The groups were compared by means of Kruskal-Wallis tests and, subsequently, Mann-Whitney tests. Neither SNAP-IV nor CPRS-HI scores showed significant differences between CG and any of the other groups. ATENTO (parents’ reports) showed no significant differences between the CG and the other groups.

ATENTO (self-report) scores showed a significant difference between the TSTM and CG groups in “school context” (*P*<.10). Additionally, the TC group showed a statistically significant difference with the CG in “time orientation” measures. None of the other comparisons reached statistical significance.

BarOn EQ-i:YV scores showed statistically significant differences between the TSTM and CG groups in “positive impression” on the interpersonal scales (*P*<.10). None of the remaining comparisons reached statistical significance.

CPT-3 scores showed differential improvements in “block change” and “inter stimulus change” in the TSTM group compared with the control group and differences in improvements in “block change” between the TC and control groups (*P*<.10). None of the other comparisons reached statistical significance.

## Discussion

### Principal Findings

This prospective, unicentric, randomized study in clinically drug-stable children with ADHD did not confirm our hypothesis that using an augmentation strategy (either TSTM or TC) may improve executive functions as measured by the BRIEF-2 total scores. An exception was the improvement in emotional control and regulation as measured by the BRIEF-2 in the TC group. Furthermore, significant pre-post symptom reductions were found for several scales measuring secondary end points, and they differ depending on the type of intervention (as explained in the following sections).

### TSTM

We found no improvements in executive functioning after using TSTM. This finding is consistent with literature using other serious video games such as ACTIVATE [60] or Plan-It Commander [61]. However, this last video game demonstrated improvements in working memory and planification skills, which have not been observed in our study. In addition, a recent review found that some video games may be beneficial for executive function training in patients with ADHD [62].

However, we found that the TSTM group showed better posttreatment scores in core ADHD symptoms, in particular inattentive symptoms, compared to the CG, mainly in self-reported tests. Moreover, parents reported improvements in social and school skills, consistent with results coming from studies using other serious video games like Plan-It Commander [61] and ThinkRx [63].

But the most relevant finding is that the TSTM group, compared to the CG, showed some improvement in some emotional domains. Both self-reports and parent reports provided better scores on scales related to emotional intelligence, emotional regulation, and performance in school context compared to the CG, which is in keeping with some other literature [64-67]. Better than a purely cognitive tool, the TSTM video game may serve as a way to train and improve self-regulation and social skills through facing challenging problems and competitions with a social component.

### TC

As was the case with TSTM, we did not find any improvement in the TC group when compared with the CG, with the remarkable exception of emotional control and regulation. This negative finding is in contradiction with several studies reporting improvements in executive functioning using a therapy based on board games (eg, chess and Go) [31]. Demily et al [68] reported that patients with schizophrenia who played chess for just 10 sessions improved executive functioning (*z*=–3.41). Finally, a recent systematic review and meta-analysis suggested that some of these board games may improve self-efficacy, attitudes, social functioning, and executive functions [69]. Our negative findings might be explained either by differences in the studied populations (the studies were devoted to patients with diagnoses apart from ADHD) or that TC was implemented electronically.

However, core ADHD symptoms benefited from TC. In particular, inattentive symptoms improved most in the TC group when compared with the CG. This result is consistent with that obtained in a pilot study using chess in children with ADHD [28]. Furthermore, as was the case with TSTM, the greatest improvements obtained by TC training were in the emotional and interpersonal spheres. Significantly better improvements were shown in emotional intelligence, emotional regulation and control, as well as interpersonal scales, as measured by the BRIEF-2 and BarOn EQ-i:YV scores. Our findings are in keeping with previous studies reporting that chess training has shown good results in emotional intelligence development [30,70].

### Strengths and Limitations

The main strength of this study is the use of 2 gaming strategies for treating ADHD, following an add-on strategy with clinically stable patients; one of them was disruptive—the use of a serious video game (TSTM)—and the other (TC) was more traditional, thus allowing for testing the effectiveness of each tool in executive functions and ADHD core symptoms. In contrast, the main limitation was the irruption of the COVID-19 pandemic, which impacted the clinical trial, as some patients randomized to the TSTM group had more difficulty completing the cognitive training sessions. Another limitation was the use of just one TSTM session per week in the TSTM group, which may have limited the effectiveness of this cognitive training. Accordingly, we plan to include a 2-3 session-per-week strategy in the next clinical trial using TSTM, scheduled to begin in April 2023. Furthermore, the evaluation was not blind and was based on clinical judgment and either self-report or parental reports. However, previous work states that teachers, rather than parents, tend to be the most reliable external observers of ADHD symptoms in children [71]. Furthermore, we used the CPT-3 to have an objective measure of our results. Another limitation was the difference between the ATI and the PP populations. Samples sizes for the groups were not very different, but a potential survival bias may have conditioned our results. It is possible that adherence to treatment was conditioned by the differential attractiveness of the video game and TC, that is, children who like video games or chess were more likely to complete the trial, and that could bias the results for the PP population. Indeed, in the PP analyses, fewer patients finished the TC intervention in the TC group. It is possible that only those patients with ADHD who were motivated or who liked chess finished the treatment, thus biasing our results in the TC arm. Another difference is the alternate location of the TSTM treatment, administered in our hospital, compared to the TC treatment, self-administered at the patients’ homes. Moreover, a possible limitation is the use of drug-stable samples; perhaps the effects of playing serious video games or TC may be more prominent in patients without a stable pharmacological treatment. Furthermore, to have clinical samples as representative and general as possible, we recruited participants in a wide age range, but our samples were not large enough to allow for analyses across developmental stages; future research should study in depth the differential effects across ages. Finally, the lack of a long-term follow-up approach kept us from studying the maintenance of any beneficial effect over time.

### Conclusions

Neither TSTM nor TC produced a significant improvement in the executive functioning of patients with ADHD who were clinically stable on ADHD drugs. However, both TSTM and TC produced a significant improvement in some ADHD symptoms, particularly in emotional regulation areas. Further studies are warranted to extend these preliminary but motivating findings.

## Appendix

Multimedia Appendix 1 Descriptive baseline characteristics.

Multimedia Appendix 2 CONSORT e-Health checklist.

## Abbreviations

ADHD: attention-deficit/hyperactivity disorder
ATI: analyses for treatment intention
BRIEF-2: Behavior Rating Inventory of Executive Function-2
CG: control group
CPRS: Conners’ Parent Rating Scale
CPT-3: Continuous Performance Test 3
EQ-i: YV Emotional Quotient Inventory: Youth Version
PP: per protocol
TC: Therapeutic Chess
TSTM: The Secret Trail of Moon
UKU: Udvalg für Kliniske Undersolgester
