# Supplementary material for: Effectiveness of a Personalized, Chess-Based Training Serious Video Game in the Treatment of Adolescents and Young Adults With Attention-Deficit/Hyperactivity Disorder: Randomized Controlled Trial
Source: JMIR Serious Games. 2023 Apr 24;11:e39874. doi: 10.2196/39874 (PMC10167585; doi:10.2196/39874)
Supplement: Multimedia Appendix 1 [file games_v11i1e39874_app1.docx]

Table S1: Baseline characteristics for the three groups

| Test | Scale | Statistic(s) | TSTM (n=35) | TC (n=34) | CG (n=35) |
| --- | --- | --- | --- | --- | --- |
| BRIEF-2 | Inhibition | Percentile 50 (25; 75) | 16 (11; 19) | 16 (13; 18) | 16 (12; 19) |
|  | Self-supervision | Percentile 50 (25; 75) | 9 (7; 7) | 8.5 (7; 11) | 8 (6; 10) |
|  | Flexibility | Percentile 50 (25; 75) | 17 (14; 19) | 17 (14; 21) | 17 (14; 21) |
|  | Emotional control | Percentile 50 (25; 75) | 16 (10.5; 19.5) | 16 (13.25; 20.75) | 16 (12; 18) |
|  | Initiative | Percentile 50 (25; 75) | 11 (9; 11.5) | 12 (10; 12.75) | 12 (9; 13) |
|  | Working Memory | Percentile 50 (25; 75) | 16 (14; 19) | 18 (16; 20.75) | 19 (16; 22) |
|  | Planning / Organization | Percentile 50 (25; 75) | 17 (16; 20.5) | 20 (16.25; 22) | 20 (17; 22) |
|  | Task supervision | Percentile 50 (25; 75) | 11 (8.5; 13) | 12 (9.25; 14) | 12 (10; 13) |
|  | Material organization | Percentile 50 (25; 75) | 12 (9; 14) | 11.5 (10; 15) | 13 (11; 15) |
| SNAP | Inattentive | Percentile 50 (25; 75) | 14 (9; 19) | 17.50 (1; 22) | 18 (11; 21) |
|  |  | Diag. Inattentive (> 15) | 11 (31.43%) | 18 (52.94%) | 20 (57.14%) |
|  | Hyperactive/Inattentive | Percentile 50 (25; 75) | 10 (4; 14) | 10.5 (3; 16) | 11 (7; 18) |
|  |  | Diag. Hyperactive (> 16) | 4 (11.43%) | 7 (23.59%) | 10 (28.57%) |
| CPRS-HI |  | Percentile 50 (25; 75) | 13 (10; 16) | 15.5 (8; 19) | 15 (10; 19) |
|  |  | Diag. ADHD (> 12) | 19 (54.29%) | 20 (58.82%) | 20 (57.14%) |
| ATENTO (Parents) | Inattention | Percentile 50 (25; 75) | 63 (56; 74) | 69 (59; 74) | 68 (63; 76) |
|  | Hyperactivity / Impulsivity | Percentile 50 (25; 75) | 61 (48; 67) | 59.5 (50; 71) | 61 (51; 72) |
|  | Attentional Control | Percentile 50 (25; 75) | 61 (57; 71) | 67 (59; 75) | 66 (63; 72) |
|  | Behavioral Regulation | Percentile 50 (25; 75) | 61 (50; 78) | 61 (50; 72) | 62 (53; 72) |
|  | Emotional Regulation | Percentile 50 (25; 75) | 68 (55; 73) | 64.5 (57; 79) | 66 (57; 76) |
|  | Working Memory | Percentile 50 (25; 75) | 64 (55; 75) | 61.5 (57; 76) | 69 (64; 78) |
|  | Flexibility | Percentile 50 (25; 75) | 71 (58; 75) | 66.5 (57; 81) | 70 (63; 79) |
|  | Planning / Organization | Percentile 50 (25; 75) | 66 (59; 72) | 68.5 (60; 74) | 69 (63; 76) |
|  | Time Orientation | Percentile 50 (25; 75) | 61 (52; 71) | 59 (53; 70) | 67 (56; 76) |
|  | Behavioral Problems | Percentile 50 (25; 75) | 55 (47; 61) | 51.5 (46; 72) | 52 (47; 62) |
|  | Sleeping Problems | Percentile 50 (25; 75) | 55 (44; 60) | 53 (45; 63) | 58 (49; 68) |
| EQ-i:YV | Positive Impression | Percentile 50 (25; 75) | 96 (76; 105) | 96 (83; 109) | 100 (87; 116) |
|  | Mood | Percentile 50 (25; 75) | 96 (78; 109) | 95 (81; 109) | 98 (81; 116) |
|  | Total Emotional Intelligence | Percentile 50 (25; 75) | 98 (88;105) | 97 (86; 108) | 103 (84; 113) |
|  | Intrapersonal | Percentile 50 (25; 75) | 97 (91; 108) | 97 (89; 108) | 97 (89; 110) |
|  | Interpersonal | Percentile 50 (25; 75) | 99 (86; 109) | 95.5 (87; 106) | 110 (100; 118) |
|  | Adaptability | Percentile 50 (25; 75) | 102 (84; 112) | 90 (80; 106) | 99 (81; 111) |
|  | Stress Management | Percentile 50 (25; 75) | 104 (95; 111) | 99.5 (87; 109) | 99 (89; 107) |
| CPT-3 | Response Style | Percentile 50 (25; 75) | 51 (43; 58) | 49.5 (45; 57) | 52 (45; 58) |
|  | Detectability | Percentile 50 (25; 75) | 46 (38; 53) | 47 (37; 55) | 46 (42; 51) |
|  | Omissions | Percentile 50 (25; 75) | 45 (43; 48) | 45 (44; 49) | 45 (43; 48) |
|  | Comissions | Percentile 50 (25; 75) | 45 (40; 51) | 50 (39; 56) | 46 (39; 52) |
|  | Perseverations | Percentile 50 (25; 75) | 48 (45; 54) | 47.5 (45; 51) | 46 (45; 50) |
|  | Reaction time | Percentile 50 (25; 75) | 51 (46; 57) | 51 (47; 58) | 53 (47; 58) |
|  | Reaction time standard deviation | Percentile 50 (25; 75) | 47 (42; 52) | 47 (45; 53) | 46 (40; 54) |
|  | Variability | Percentile 50 (25; 75) | 45 (42; 52) | 46 (42; 51) | 46 (41; 50) |
|  | Block change | Percentile 50 (25; 75) | 49 (45; 55) | 51.5 (46; 57) | 53 (49; 60) |
|  | Interstimulus change | Percentile 50 (25; 75) | 50 (41; 57) | 48 (42; 57) | 49 (45; 57) |

Table S2: Characteristics of the three samples after treatment

| Test | Scale | Statistic(s) | TSTM (n=35) | TC (n=34) | CG (n=35) |
| --- | --- | --- | --- | --- | --- |
| BRIEF-2 | Inhibition | Percentile 50 (25; 75) | 14 (11.75; 18) | 14.50 (9.25; 18) | 15 (12; 19) |
|  | Self-supervision | Percentile 50 (25; 75) | 8 (7; 10) | 8 (6; 9.75) | 8 (7; 11) |
|  | Flexibility | Percentile 50 (25; 75) | 16 (12.75; 19) | 17 (12.5; 21.75) | 17 (14; 19) |
|  | Emotional control | Percentile 50 (25; 75) | 15 (11; 18) | 15 (12; 17) | 15 (12; 19) |
|  | Initiative | Percentile 50 (25; 75) | 10 (9; 12) | 11 (10; 13) | 11 (10; 13) |
|  | Working Memory | Percentile 50 (25; 75) | 17.5 (13; 19) | 16.5 (14.25; 22) | 19 (15; 21) |
|  | Planning / Organization | Percentile 50 (25; 75) | 17 (15.75; 19) | 19 (16; 21) | 19 (16; 22) |
|  | Task supervision | Percentile 50 (25; 75) | 11 (9.75; 12.25) | 11 (9.25; 13.75) | 12 (10; 13) |
|  | Material organization | Percentile 50 (25; 75) | 11.5 (8.75; 14.25) | 12 (9.5; 15) | 13 (10; 15) |
| SNAP | Inattentive | Percentile 50 (25; 75) | 13 (8; 16) | 13 (11; 18) | 17 (14; 21) |
|  | Hyperactive/Inattentive | Percentile 50 (25; 75) | 9 (4; 13) | 7 (3; 16) | 11 (5; 17) |
| CPRS-HI |  | Percentile 50 (25; 75) | 12 (7; 15.5) | 12 (6.25; 18.75) | 14 (9; 19) |
| ATENTO (Parents) | Inattention | Percentile 50 (25; 75) | 66.5 (55; 75) | 70.5 (61.5; 82,5) | 73 (57; 84) |
|  | Hyperactivity / Impulsivity | Percentile 50 (25; 75) | 42.5 (33.5; 51.25) | 38.5 (28.5; 55.75) | 48 (34; 60) |
|  | Attentional Control | Percentile 50 (25; 75) | 47.5 (40.75; 54) | 50 (42.5; 61.75) | 53 (43; 63) |
|  | Behavioral Regulation | Percentile 50 (25; 75) | 52 (40.5; 61.25) | 48 (36.5; 68.75) | 57 (40; 73) |
|  | Emotional Regulation | Percentile 50 (25; 75) | 38.5 (29; 44.5) | 41.5 (29.75; 48.75) | 40 (30; 51) |
|  | Working Memory | Percentile 50 (25; 75) | 39 (27; 49) | 43.5 (34; 52.5) | 44 (36; 55) |
|  | Flexibility | Percentile 50 (25; 75) | 40 (33; 51) | 47.5 (37; 52.75) | 45 (39; 54) |
|  | Planning / Organization | Percentile 50 (25; 75) | 64 (54.75; 72.75) | 76 (55.25; 81.75) | 72 (54; 86) |
|  | Time Orientation | Percentile 50 (25; 75) | 20 (15; 25.25) | 19.5 (16; 26.75) | 25 (15; 29) |
|  | Behavioral Problems | Percentile 50 (25; 75) | 20 (16; 29) | 24.5 (19; 35) | 21 (17; 30) |
|  | Sleeping Problems | Percentile 50 (25; 75) | 15 (10.75; 20,25) | 18.5 (12; 19.75) | 18 (12; 24) |
| EQ-i:YV | Positive Impression | Percentile 50 (25; 75) | 14 (13; 16.25) | 14 (13; 16) | 14 (12; 16) |
|  | Mood | Percentile 50 (25; 75) | 43 (38; 48.5) | 45 (39; 49.75) | 45 (37; 50) |
|  | Total Emotional Intelligence | Percentile 50 (25; 75) | 54 (50.25; 61) | 52.5 (49; 58) | 54 (48; 60) |
|  | Intrapersonal | Percentile 50 (25; 75) | 14 (9.75; 16) | 13 (11; 15.75) | 13 (10; 16) |
|  | Interpersonal | Percentile 50 (25; 75) | 38.5 (35; 45) | 38 (33.25; 42.5) | 39 (36; 43) |
|  | Adaptability | Percentile 50 (25; 75) | 28 (22.75; 33.25) | 25 (22; 29) | 26 (22; 29) |
|  | Stress Management | Percentile 50 (25; 75) | 31 (28; 33.35) | 30.5 (25.25; 35.75) | 32 (27; 34) |
| CPT-3 | Response Style | Percentile 50 (25; 75) | 57.50 (50.75; 61.5) | 51 (45; 68.75) | 52 (48; 62) |
|  | Detectability | Percentile 50 (25; 75) | 46.5 (40.25; 56.25) | 43 (35.5; 48.25) | 45 (35; 52) |
|  | Omissions | Percentile 50 (25; 75) | 47 (43; 51.25) | 44.5 (43; 48.5) | 45 (43; 49) |
|  | Comissions | Percentile 50 (25; 75) | 44 (36.75; 54) | 43 (35; 51) | 43 (38; 51) |
|  | Perseverations | Percentile 50 (25; 75) | 48 (45; 57) | 45.5 (44; 48) | 46 (45; 51) |
|  | Reaction time | Percentile 50 (25; 75) | 52.5 (47.75; 57.25) | 52.5 (45.25; 64.75) | 52 (45; 65) |
|  | Reaction time standard deviation | Percentile 50 (25; 75) | 49.5 (41; 61.25) | 45 (41.25; 51.5) | 46 (41; 53) |
|  | Variability | Percentile 50 (25; 75) | 45.5 (43.75; 58.5) | 42.5 (41; 49) | 45 (42; 52) |
|  | Block change | Percentile 50 (25; 75) | 51 (46; 56.25) | 55.5 (48.25; 60.75) | 52 (45; 57) |
|  | Interstimulus change | Percentile 50 (25; 75) | 51.5 (45.75; 60) | 49 (43.5; 60) | 49 (45; 59) |
